# Plackett-Burman Design for rGILCC1 Laccase Activity Enhancement in* Pichia pastoris*: Concentrated Enzyme Kinetic Characterization

**DOI:** 10.1155/2017/5947581

**Published:** 2017-03-21

**Authors:** Edwin D. Morales-Álvarez, Claudia M. Rivera-Hoyos, Ángela M. Cardozo-Bernal, Raúl A. Poutou-Piñales, Aura M. Pedroza-Rodríguez, Dennis J. Díaz-Rincón, Alexander Rodríguez-López, Carlos J. Alméciga-Díaz, Claudia L. Cuervo-Patiño

**Affiliations:** ^1^Laboratorio de Microbiología Ambiental y de Suelos, Grupo de Biotecnología Ambiental e Industrial (GBAI), Departamento de Microbiología, Facultad de Ciencias, Pontificia Universidad Javeriana (PUJ), Bogotá, Colombia; ^2^Departamento de Química, Facultad de Ciencias Exactas y Naturales, Universidad de Caldas, Manizales, Caldas, Colombia; ^3^Laboratorio de Biotecnología Molecular, Grupo de Biotecnología Ambiental e Industrial (GBAI), Departamento de Microbiología, Facultad de Ciencias, Pontificia Universidad Javeriana (PUJ), Bogotá, Colombia; ^4^Laboratorio de Expresión de Proteínas, Instituto de Errores Innatos del Metabolismo (IEIM), Facultad de Ciencias, Pontificia Universidad Javeriana (PUJ), Bogotá, Colombia; ^5^Laboratorio de Parasitología Molecular, Grupo de Enfermedades Infecciosas, Facultad de Ciencias, Pontificia Universidad Javeriana (PUJ), Bogotá, Colombia

## Abstract

Laccases are multicopper oxidases that catalyze aromatic and nonaromatic compounds with concomitant reduction of molecular oxygen to water. They are of great interest due to their potential biotechnological applications. In this work we statistically improved culture media for recombinant GILCC1 (rGILCC1) laccase production at low scale from* Ganoderma lucidum* containing the construct pGAPZ*α*A-*GlucPost*-Stop in* Pichia pastoris*. Temperature, pH stability, and kinetic parameter characterizations were determined by monitoring concentrate enzyme oxidation at different ABTS substrate concentrations. Plackett-Burman Design allowed improving enzyme activity from previous work 36.08-fold, with a laccase activity of 4.69 ± 0.39 UL^−1^ at 168 h of culture in a 500 mL shake-flask. Concentrated rGILCC1 remained stable between 10 and 50°C and retained a residual enzymatic activity greater than 70% at 60°C and 50% at 70°C. In regard to pH stability, concentrated enzyme was more stable at pH 4.0 ± 0.2 with a residual activity greater than 90%. The lowest residual activity greater than 55% was obtained at pH 10.0 ± 0.2. Furthermore, calculated apparent enzyme kinetic parameters were a *V*_max_ of 6.87 × 10^−5^ mM s^−1^, with an apparent *K*_*m*_ of 5.36 × 10^−2^ mM. Collectively, these important stability findings open possibilities for applications involving a wide pH and temperature ranges.

## 1. Introduction

Laccases are blue multicopper oxidases (EC 1.10.3.2), catalyzing oxidation reactions for an array of compounds such as diphenols, polyphenols, deamines, aromatic amines, inorganic compounds, and nonphenolic compounds in the presence of redox mediators. During the reaction the substrate is oxidized by donating its electron, where molecular oxygen acts as an electron acceptor and is reduced into water [1–3].

Laccases are enzymes mainly produced in white rot fungi; however they are widely distributed in plants, insects, fungi, and bacteria [1, 4–6]. They have important applications in different industrial settings, as they help to reduce the environmental impact of their waste. These applications include dye bleaching in textile industry, bleaching of cellulose pulp, detoxification of residual waters, toxic compound bioremediation, biosensor construction, fuel cells, fruit juice processing, and synthesis of molecules in the pharmaceutical industry [3, 6–10].

Laccase biotechnological and environmental applications require great enzyme quantities; unfortunately laccases obtained from natural sources are not suitable for long growth periods, low product/biomass (*Y*_*p*/*x*_) or product/substrate (*Y*_*p*/*s*_) yield, and prolonged, complex, and costly isolation procedures [3, 6, 11]. Therefore, heterologous expression is a promising option for greater scale production, using the potential of hosts that are easy to handle and culture, such as bacteria and yeast [12].

Yeast offers fast growth rates, ease of gene manipulation, and posttranslational modification capabilities.* P. pastoris *has been employed for years as an industrial platform for heterologous protein expression. Moreover, it is one of the most effective expression systems to obtain high yield extracellular proteins [14–17]. Additionally, various reports have described better expression and productivity levels thorough culture media optimization [4, 18].

The objective of this work was to increase* Ganoderma lucidum *rGILCC1 laccase activity in* Pichia pastoris *at low scale in the laboratory to determine pH and temperature stability and define its *V*_max_ and *K*_*m*_ in concentrated supernatant obtained from microbial culture. This was achieved by improving the following factors: nitrogen and source type (organic and inorganic), carbon concentration (glucose), copper concentration, oxygen transfer (media volume/Erlenmeyer flask volume ratio), time of culture, and inoculum percentage.

## 2. Materials and Methods

### 2.1. Strain

 The strain was* P. pastoris* X33 containing pGAPZ*α*A-*LacGluc-Stop* (Clone 1) expression vector with previously optimized synthetic gene* GILCC1 *coding for* Ganoderma lucidum *GILCC1 laccase. This strain was kept in 1% YPD (w/v), 2% peptone, 1% yeast extract, and 2% D+ glucose, supplemented with 20% glycerol (w/v), and kept at −80°C [19–21].

### 2.2. Inoculum Preparation

*Pichia pastoris X33/pGAPZαA-LaccGluc-Stop* clone 1 Master Cell Bank (MCB) [21] was thawed and used for inoculating 5 mL screw cap tubes with sterile YPD supplemented with 40 *µ*g mL^−1^ zeocin (Z). Tubes were incubated overnight (ON) at 30°C with 180 rpm agitation, followed by inoculation under the same conditions for 12 h in 500 mL Erlenmeyer flasks, containing 100 mL (effective work volume) of fresh YPD-Z. The resulting culture was verified by Gram stain to detect presence of contaminating morphologies and used for factorial design inoculations.

### 2.3. Plackett-Burman Experimental Design (PBED)

Seven factors were evaluated with two levels each as follows: media volume (150 and 300 mL), CuSO_4_ concentration (0.1 and 1.0 mM), inoculum percentage (2 and 10% (v/v)), glucose concentration (10 and 30 gL^−1^), NH_4_SO_4_ concentration (5 and 20 mM), peptone concentration (10 and 20 gL^−1^), and yeast extract concentration (5 and 10 gL^−1^). The design included a central point evaluated three times; values within central points were 225 mL media, 0.55 mM CuSO_4_, 6% inoculum (v/v), 20 gL^−1^ glucose, 12.5 mM NH_4_SO_4_, 15 gL^−1^ peptone, and 7.5 gL^−1^ yeast extract [22]. For statistical analysis the response variable evaluated was enzyme activity (UL^−1^). Additionally, to determine if statistically significant differences were observed among treatments (*T*_1_–*T*_12_) a one-way ANOVA with Tukey post hoc test was performed, employing a 95% confidence interval (CI, *α* = 0.05). Moreover, Shapiro-Wilk normalization test was applied to verify data quality using SAS V 9.0® 2004 (SAS Institute Inc., Cary, NC, USA).

*Note*. Enzyme activity (UL^−1^), protein concentration (mg mL^−1^), and glucose concentration (gL^−1^), specific activity (UL^−1^ mg mL^−1^), and productivity (UL^−1^h^−1^) were assayed 0 to 12 h every two hours. The same variables were then evaluated every 24 h up to the end (168 h), since preliminary data revealed that better results were obtained between 156 h and 168 h [19]. Enzyme activity (UL^−1^) was the response variable utilized for statistical analyses. All improvement assays were carried out in 500 mL Erlenmeyer flasks at 30°C and 180 rpm, employing the same flask for the total 168 h of culture at variable pH starting at 7 ± 0.2. Design Expert V. 9.0 (Stat-Ease, Inc., Minneapolis, MN, USA) software was used to devise experimental design and result analysis. In addition, Sigma Plot V.11.0 software (Systat Software Inc. San José, CA, USA) was employed to graph concentrated enzyme kinetics and results.

### 2.4. Supernatant Concentrate

Culture supernatant demonstrating the highest enzymatic activity values with ABTS substrate [2,20-azino-bis(3-ethylbenzothiazoline-6-sulphonic acid)] under conditions previously described [23] (17.9 mg mL^−1^ protein and 4.69 ± 0.39 UL^−1^ enzyme activity, with a specific enzyme activity of 0.26 Umg^−1^ at 168 h of culture) was used. Briefly, culture was centrifuged at 4°C 8,000*g* and supernatant was filtered in a serial manner through Whatman Number 1 filter paper, followed by 0.45 and 0.22 *µ*m membranes (Pall Corp, Port Washington, NY, USA). The filtrate was concentrated by centrifugation employing a 10 kDa Ultracel regenerated cellulose membrane (Millipore, Billerica, MA, USA) [23]. Approximately 20 mL concentrate was used to perform the enzyme's functional identity by zymography.

### 2.5. Enzyme Functional Identification

Zymogram was ran in 12% (w/v) native PAGE under nondenaturing conditions. Activity or functionality was visualized by 0.5 M ABTS stain. BenchMark™ Pre-Stained Protein ladder (Life Technologies™, USA) was used as the molecular weight standard and Lac® (Sigma-Aldrich®, St. Louis, MO, USA) as laccase control.

### 2.6. Temperature Stability

Concentrated enzyme temperature stability was assayed by incubating for 1 h at the following temperatures: 10, 20, 30, 40, 50, 60, and 70°C; subsequently residual enzyme activity was determined under standard assay conditions [13]. All assays were performed at least three times.

### 2.7. pH Stability

To establish pH stability supernatant obtained from concentrate was previously incubated for 1 h at 25°C in the absence of substrate using Britton-Robinson buffer [24] with pH values ranging between 2 and 12 ± 0.2, followed by laccase residual enzyme activity determination under standard assay conditions [13]. All assays were performed at least three times.

### 2.8. Kinetic Constant

Concentrated enzyme kinetic constants were evaluated using ABTS as substrate in a concentration range between 0.05 and 0.5 mM at 0.1 mM intervals in 600 mM sodium acetate buffer at pH 4.5. For all assays 800 *µ*l concentrated enzyme with 4.49 UL^−1^ enzyme activity was employed at 25°C. After hyperbola adjustment using Michaelis-Menten equation *V*_max_ and apparent *K*_*m*_ were calculated following Hanes-Woolf linearization method [25], with the aid of SIMFIT software V5.40, 2003 (W.G. Bardsley, University of Manchester, UK) [26]. All kinetic assays were performed at least three times.

### 2.9. Determination of Total Residual Reducing Sugar Concentration

3,5-Dinitrosalicylic acid colorimetric method (DNS) was employed to evaluate total residual reducing sugars [27] for each sample (in triplicate). A 0.1 and 2 g L^−1^ D-glucose curve was used as the standard.

### 2.10. Total Extracellular Protein Concentration Determination

Total extracellular protein concentration was established by Biuret [28] methodology for each sample (in triplicate). A bovine serum albumin (BSA) curve between 0.5 and 5 mg mL^−1^ was used as a standard curve.

### 2.11. Enzyme Activity Quantification

Enzyme activity was monitored by changes in absorbency at 436 nm (*ε*_436_ = 29,300 M^−1^cm^−1^) as a result of ABTS in a 60 mM (pH 4.5 ± 0.2) sodium acetate buffer. 100 *µ*L 5 mM ABTS as substrate, 800 *µ*L crude extract at room temperature (RT), and 100 *µ*L 600 mM sodium acetate buffer were used. Formation of a green cationic radical was evaluated spectrophotometrically for three minutes. A unit of activity is defined as the quantity of enzyme required to oxidize 1 *µ*mol ABTS in one minute. Blanc solution contained 800 *µ*L distilled water, 100 *µ*L 600 mM sodium acetate buffer, and 100 *µ*L 5 mM ABTS. Enzyme activity was expressed in UL^−1^ [13].

Specific activity was calculated by dividing the obtained enzyme activity for each hour of culture by total protein concentration and expressed in Umg^−1^:

(1) $$\begin{array}{c} \text{Spec. Act.}\frac{\text{Enz. Act.}}{\text{Prot. Conc.}}, \end{array}$$

where enzyme activity (enz. act.) is given in UL^−1^ and protein concentration (prot. conc.) in mg mL^−1^.

Productivity in function of enzyme activity was expressed as biological activity UL^−1^ h^−1^ (see (2)), calculated in the following manner:

(2) $$\begin{array}{c} P_{Enz.}=\frac{\text{Enz. Act.}}{\text{Time}}. \end{array}$$

## 3. Results

### 3.1. Plackett-Burman Experimental Design (PBED)

ANOVA for a model that was not adjusted for curvature was significant (*p* = 0.0034), allowing for evaluation of the different factors'* “main effect”* on culture extracellular enzyme activity detected at 168 h.  Table 1 depicts model's significant values for each factor involved. Polynomial equation (3) represents laccase activity and can be used for predictions based on different evaluated levels for each factor. Additionally, the equation can also result as being useful for factor relative impact identification when comparing obtained coefficients between them

(3) Enz. Act.168 h=0.79−0.31×A+0.52×B+0.11×D+0.10×E+0.44×F−0.48×G−0.84×AB−0.71×AD+0.60×AE+0.82×AG.

**Figure 1:**
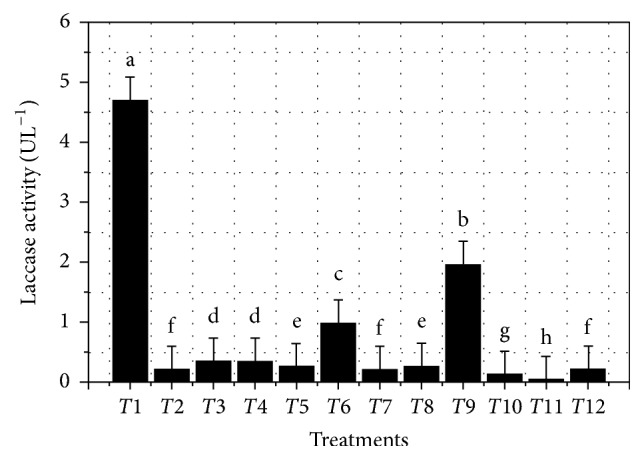
PBED mean ± SD treatment results. Each treatment was assayed in triplicate (*n* = 3). Means ± SD were compared among all twelve treatments.* p* < 0.05 was significant.

Enzyme activity for each treatment is shown in Table 2 and Figure 1, highlighting the best treatments (*T*_1_ and *T*_9_), with enzyme activity values greater than 1.8 UL^−1^, as well as mean comparison among the 12 treatments.

For most treatments (Figure 1 and Table 2) enzyme activity exceeded that obtained from previous work at 156 h (>0.13 ± 0.03 UL^−1^). *T*_1_ and *T*_9_ treatments were the most significant, hence promising, with *T*_1_ attaining after 168 h of culture the highest enzyme activity 4.69 ± 0.39 UL^−1^. This represents an approximate 36.08-fold increase in comparison with that obtained from previous work [19].

Laccase activity presented a great variation among the 12 treatments (Table 2), evidencing the relevance and usefulness of culture media improvement. In addition, it is important to note that none of the predictions exceeded the results obtained from treatment *T*_1_.

Positive and negative effects are shown in Table 3 and each factor is involved in PBED percentage contribution on the response variable (enzyme activity UL^−1^). As can be observed from Table 1 and (3), analysis hierarchy discarded Factor* C*, inoculum.

**Figure 2:**
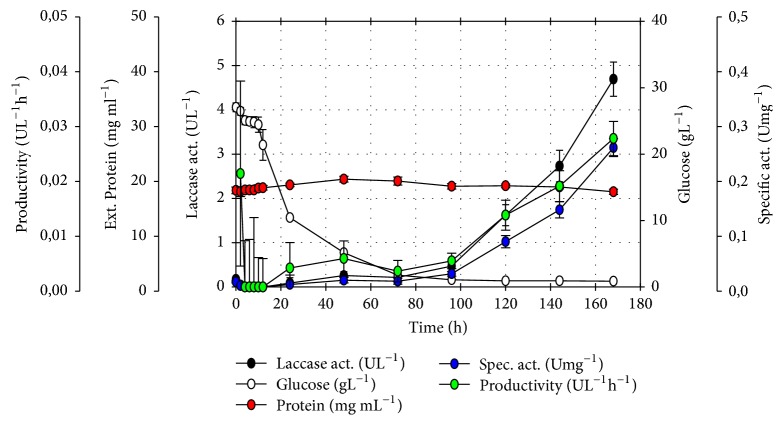
Treatment *T*_1_ PBED kinetic follow-up. Treatment 1 [PBED-*T*_1_: 500 mL Erlenmeyer flask containing 150 mL media, 10% inoculum (v/v), 1.0 mM CuSO_4_, 30 gL^−1^ glucose, 5 mM NH_4_SO_4_, 10 gL^−1^ peptone, and 5 gL^−1^ yeast extract], enzy. act. 4.69 ± 0.39 UL^−1^ at 168 h of culture. Assay was carried out in triplicate (*n* = 3).

PBED kinetic follow-up for *T*_1_ revealed the highest productivity (UL^−1^ h^−1^) based on enzymatic activity. Enzyme activity (UL^−1^) and specific activity (Umg^−1^) were obtained after 168 h of culture (Figure 2).

### 3.2. Concentrated Enzyme Functional Identification

**Figure 3:**
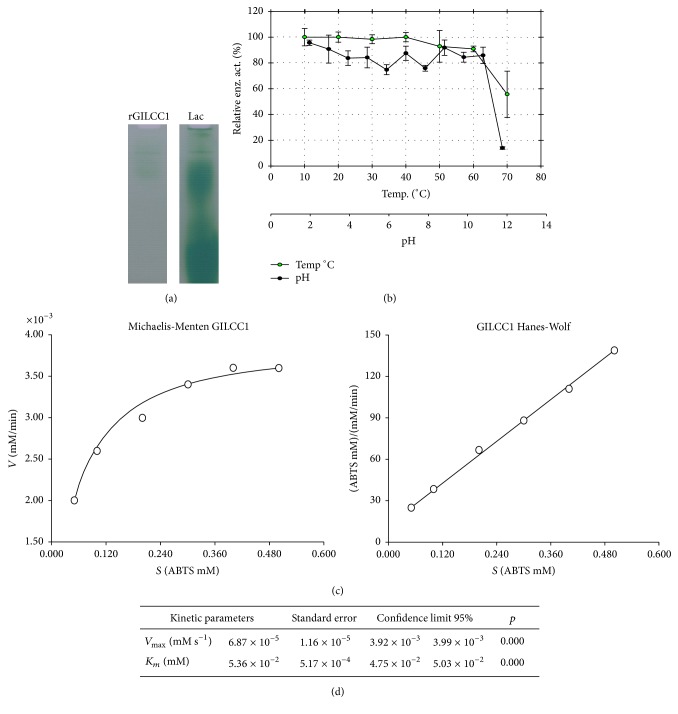
rGILCC1 concentrate characterization. (a) Zymogram gel for rGILCC1 enzyme functional identification. As positive control,* Laccase* Lac* (Sigma-Aldrich)*. (b) Relative enzyme activity (%) as a function of rGILCC1 obtained from concentrate after 1 h incubation at different temperatures and pH (assay carried out in triplicate,* n* = 3). Activity was determined as described in Materials and Methods [13]. (c) rGILCC1 obtained from concentrate enzyme kinetics using ABTS as a substrate (assay carried out in triplicate,* n* = 3). (d) *K*_*m*_ (Michaelis-Menten constant), *V*_max_ (maximal velocity) detailed values.

 The functional identification of rGILCC1 enzyme by zymogram (native PAGE) using 0.5 M ABTS in 60 mM sodium acetate buffer stain is shown in Figure 3(a). Commercial laccase presented various active fractions, suggesting the positive control as a possible multimeric laccase (Figure 3(a)).

### 3.3. Temperature and pH Stability

 rGILCC1 relative enzyme activity (%) temperature and pH stability results obtained from concentrate after incubating the concentrate for 1 h at different temperatures or 25°C at different pHs are shown in Figure 3(b). As was observed enzyme activity between 10 and 60°C was greater than 80%. For pH ranges between pH 2 and pH 11 enzyme activity ranged between 75 and 100%.

### 3.4. Kinetic Constants

rGILCC1 laccase obtained from concentrate kinetic characteristics for ABTS oxidation is shown in Figure 3(c), where *V*_max_ values under assay conditions (*V*_max_ = 6.87 × 10^−5^ mMs^−1^) and Michaelis-Menten constant (*K*_*m*_ = 5.36 × 10^−2^ mM) were also estimated. Hanes-Woolf model best described enzyme concentrate behavior, under assay conditions (temp. 25°C; pH 4.5 ± 0.2).

## 4. Discussion

As can be seen in Table 1 the hierarchical model was significant, as well as *A*, *B*, *F*, and *G* factors. In this regard, at 168 h the model's *F* value was 16.17, implying that the model was significant. In contrast, there was only a 0.34% possibility of a greater *F* value due to experimental noise. A lack of adjustment *F* value of 0.14 suggests it was not significant in relation to pure error. In addition, there would be an 87.77% likelihood of a greater lack of adjustment *F* value as a consequence of noise generated in the experiments. Therefore, a nonsignificant lack of adjustment was positive for the model.

On the other hand, a predicted *R*^2^ of 0.7479 was in agreement with an adjusted *R*^2^ of 0.91, since the difference was less than 0.2. Furthermore, an adequate precision signal/noise ratio presented a 16.258 value, where a ratio greater than 4 is desirable, indicating a suitable signal. In addition, it demonstrated this model can be utilized to navigate through the design's space.

As shown in Figure 1 PBED mean ± SD comparison established treatment results were significantly different, where treatment 1 was the most prominent. Likewise, it is shown in Table 2 that the predicted model did not support obtained results in *T*_1_, despite the minimal difference.

On the other hand, Table 3 result analysis required several considerations. The factor “culture media volume” was significant (*p* = 0.0264), and a negative effect on enzyme activity was observed with a contribution percentage < 10%, implying in an optimization attempt that lower volumes could be tested. It is important to note that Erlenmeyer flaks of the same volume and brand were used; all assays were carried out in the same orbital shaker with the same* setup*, where it is clear that volume was probably associated with oxygen transfer. Decreasing the media volume would represent an increase in oxygen transfer area, which could be favorable. However, contribution percentage was small (5.81) and considered very low to propose new tests.

Furthermore, copper sulfate was also a significant factor (*p* = 0.0107) that had a positive effect on enzyme activity with a contribution percentage of 9.39%, resulting in the highest values among the factors evaluated (Table 3), suggesting that higher concentrations could be tested for optimization. On the contrary, glucose was not significant (*p* = 0.4304), despite its positive effect with a 0.44% contribution. Ammonium sulfate was not a significant factor (*p* = 0.4800) with a positive effect on enzyme activity and 0.35% contribution. Peptone factor was significant (*p* = 0.0267) with a positive effect on enzyme activity and 5.76% contribution, suggesting higher concentrations could be tested to attempt factor optimization. Last, the factor yeast extract was significant (*p* = 0.0149) with a negative effect on enzyme activity and 7.92% contribution. This result suggests lower concentrations could be tested in order to optimize this factor (Table 3).

Furthermore, contribution percentages results did not exceed 10%, and even though *A*, *B*, *D*, *E*, *F*, and *G* obtained factor values suggesting greater or lower concentrations could be assayed depending on response variable effect, carrying-out these tests would not be recommendable. Based on individual contribution percentages the change that could be generated on enzyme activity would not be substantial. Despite a 36.08-fold increase for *T*_1_ in enzyme activity in comparison with our previous work dependent variable values were still low (*T*_1_ = 4.69 ± 0.39 UL^−1^) with respect to other reported laccase activities. Additionally, Figure 2* T*_1_ time follow-up highlights that there were no higher values of the variables measured before 168 h. Therefore, the most recommendable option would be to amplify or change the navigation space when studying other conditions that perhaps would increase response variable results. Similarly other values of factors already evaluated would be studied or different factors other than the ones already assayed would be evaluated to achieve greater enzyme activity values.

On the other hand, some aspects of the results drew particular attention. Based on low glucose percentage contribution, the media could require a lower concentration of carbon source. This seems conflicting, since additional carbon would generate increased biomass. Glyceraldehyde-3-phosphate dehydrogenase (GAPDH) is critical in glycolysis. Its promoter *P*_GAP_ provides constitutive expression on glucose metabolism; therefore it has been widely used for constitutive expression of heterologous proteins. *P*_GAP_ was governing the expression of* Ganoderma lucidum* optimized laccase synthetic gene GlLCC1 [19]; thus results seem to contradict the fact that enzyme should be produced at the end of the exponential phase (Figure 2).

Our results agree with those by other authors and proposed genes under *P*_GAP_ expression are not entirely constitutive and could be regulated by additional conditions. Kern et al. (2007) studied an alternate oxidase fused to GFP under *P*_GAP_ expression. They described GFP fluorescence markedly increased after culture media glucose depletion, while in an intermitted manner small quantities of ethanol were produced, phenomena also described in other investigations [29–31]. No reports support in a detailed manner *P*_GAP_ constitutive promoter incapability of always producing the metabolite of interest in greater quantities at the end of the exponential phase. Authors argue that it is more reasonable to think of other factors associated with the intrinsic nature of the recombinant protein existing that could influence the velocity, maturation, or enzyme transport [29]. These aspects could be studied in future work in detail, whereas a case in point* Pichia pastoris* rGlLCC1 laccase expression rate and follow-up would be carried out.

Stability study demonstrated that* G. lucidum* rGILCC1 enzyme expressed in* P. pastoris* X33 was maintained stable at 10 and 60°C and retained over 50% residual enzymatic activity at 70°C (Figure 3(b)). Some authors have performed laccase GlLCC1 enzymatic stability studies, where in contrast to the present study induction of the recombinant enzyme expression in* P. pastoris *used the AOX promoter. You et al. (2014) reported a rapid decrease in enzyme stability at temperatures above 40°C [3]. Likewise, Sun et al. (2012) obtained maximal activity at 55°C; however stability tests revealed that after 20 minutes incubation at 55°C activity decreased [32] considerably [33]. Other authors have expressed the same laccase with some modifications in the N-Terminal sequence, reporting that the enzyme denatured after 10 min at 100°C incubation [34]. Moreover, after one-hour incubation at 50°C, residual activity was below 50% [32]. With respect to obtained stability in other* G. lucidum* laccases results have been variable. Manavalan et al. (2013) reported a maximal laccase-3 activity at 30°C; however, enzyme's half-life was affected even shortly before one-hour incubation at temperatures above 60°C [4]. When comparing previously mentioned pure laccase results with data obtained in this study using enzyme obtained from concentrate, we can argue that results are promising due to the ample range of thermal stability. Our laccase results are comparable or even exceeded GlLCC1 laccase stability or other* G. lucidum* laccases expressed in* P. pastoris*. These are aspects of great importance in terms of considerable potential use in different industrial settings or treatment of contaminated effluents.

rGILCC1 pH stability presented residual enzyme activities between 80 and 100% for all pHs assayed. Different authors report* P. pastoris *GlLCC1 expressed laccase great pH stability ranging between 2 and 10, obtaining relative enzyme activities above 40% for all cases [3, 4, 32, 33]. However, it is worth noting that this work evaluated up to pH 12, where residual activities of 80% were obtained. This confirms* P. pastoris *rGlLCC1 enzyme stability under the *P*_GAP_ promoter and its potential use for environmental care.

Different laccase *K*_*m*_ values, whether from fungal or bacterial origin, present an ample substrate range or may even differ from the same substrate. Nonetheless, a higher affinity to ABTS is reported in comparison to other substrates such as syringaldazine or guaiacol among others, with lower oxidation velocity and higher *K*_*m*_ values [4, 5, 35].

Our data revealed a *K*_*m*_ of 5.36 × 10^−2^ mM; this result is similar to* G. lucidum *laccase-3 [4] with a *K*_*m*_ of 0.047 mM. In contrast other studies have reported discrepant values such as Ko et al. (2001) with a *K*_*m*_ of 0.0037 mM. Other authors using GlLCC1 laccase reported superior *K*_*m*_ values as those obtained in this study with* P. pastoris X33, *namely, You et al. (2014) with 0.521 mM and Sun et al. (2012) with 0.9665 mM [3, 4, 32, 33]. However these differences could be related to laccase purity, since the enzyme used in this study is not in the pure form, hence the need to refer to it as apparent *K*_*m*_. In addition, the nature of other supernatant components is unknown or could positively or negatively interfere with enzyme activity.

## 5. Concluding Remarks

In conclusion, culture media was improved for rGILCC1 laccase production in* Pichia pastoris *[500 mL Erlenmeyer flask containing 150 mL culture media, 10% (v/v) inoculum, 1.0 mM CuSO_4_, 30 gL^−1^ glucose, 5 mM NH_4_SO_4_, 10 gL^−1^ peptone, and 5 gL^−1^ yeast extract for 168 h, at 30°C and 180 rpm] with a laccase activity of 4.7 ± 0.4 UL^−1^. This represents a 36.08-fold increase compared with our previous work. Functionality was identified from enzyme obtained from concentrate through zymogram gel. Additionally, stability at different temperatures increased to a range between 10 and 60°C. Also, at 70°C the enzyme retained 50% residual activity. Moreover, pH stability was observed between 2 and 11 with over 70% residual activity. Apparent kinetic parameters obtained by recombinant laccase demonstrated its affinity for ABTS substrate, as was reported in previous work with molecular docking analysis, and its catalytic efficiency [19], supporting what has been described for other laccases.

Additionally, it is important to note that characterization was performed from concentrated supernatant instead of pure enzyme, since the objective of this group with this and other laccases is to pave the way for liquid residue and contaminated solid treatment. Working for these purposes with pure enzyme is considerably expensive, making it unsustainable. rGILCC1 laccase can be a promising enzyme for various industries and multiple purposes due to its broad temperature and pH stability. Our next challenge is to increase culture media volume (process scale-up) in addition to augment enzyme activity, since its activity is still considered low.

## Figures and Tables

**Table 1:** Laccase activity ANOVA for a model that was not adjusted for curvature.

Source	Sum of squares	DF	Mean squares	*F*-value	*p* value
					Prob > *F*
Model	19.57	10	1.96	16.17	**0.0034**
*A, culture media volume*	*1.17*	*1*	*1.17*	*9.71*	***0.0264***
*B, CuSO*_*4*_	*1.90*	*1*	*1.90*	*15.70*	***0.0107***
*D, glucose*	*0.089*	*1*	*0.089*	*0.74*	*0.4304*
*E, NH*_*4*_ *SO*_*4*_	*0.07*	*1*	*0.07*	*0.58*	*0.4800*
*F, peptone*	*1.17*	*1*	*1.17*	*9.64*	***0.0267***
*G, yeast extract*	*1.60*	*1*	*1.60*	*13.24*	***0.0149***
Residual	0.61	5	0.12		
*Lack of fit*	*0.05*	*2*	*0.025*	*0.14*	*0.8777*
*Error*	*0.55*	*3*	*0.18*		
Cor total	20.18	15			
*R*-square	0.97				
Adjusted *R*-square	0.91				
Predicted *R*-square	0.7479				
Adequate precision	16.258				

95% significant values are in bold.

**Table 2:** PBED observed and predicted values of factors having an effect on laccase activity.

*Tx*	Factor type	Culture media volume (mL)	CuSO_4_ (mM)	Inoculum (% v/v)	Glucose (gL^−1^)	NH_4_SO_4_ (mM)	Peptone (gL^−1^)	Yeast extract (gL^−1^)	Observed enz. activity at 168 h (UL^−1^)	Predicted enz. activity at 168 h (UL^−1^)
**T**_**1**_	**Factorial**	**150**	**1**	**10**	**30**	**5**	**10**	**5**	**4.6928**	**4.6368**
*T*_2_	Factorial	300	0.1	10	30	20	10	5	0.2133	0.1182
*T*_3_	Factorial	300	1	2	30	20	20	5	0.3413	0.3635
*T*_4_	Factorial	150	1	10	10	20	20	10	0.3413	0.2853
*T*_5_	Factorial	300	0.1	10	30	5	20	10	0.2559	0.2782
*T*_6_	Factorial	150	1	2	30	20	10	10	0.9813	1.0426
*T*_7_	Factorial	150	0.1	10	10	20	20	5	0.2133	0.1573
*T*_8_	Factorial	150	0.1	2	30	5	20	10	0.2559	0.1999
**T**_9_	**Factorial**	**300**	**0.1**	**2**	**10**	**20**	**10**	**10**	**1.9625**	**1.9847**
*T*_10_	Factorial	300	1	2	10	5	20	5	0.1279	0.1502
*T*_11_	Factorial	300	1	10	10	5	10	10	0.0427	−0.0524
*T*_12_	Factorial	150	0.1	2	10	5	10	5	0.2133	0.2746
Central point		225	0.55	6	20	12.5	15	7.5	0.4693	0.7866
Central point		225	0.55	6	20	12.5	15	7.5	1.2372	0.7866
Central point		225	0.55	6	20	12.5	15	7.5	0.9386	0.7866

Best treatments are in bold.

**Table 3:** PBED evaluated factor effect and percentage contribution on laccase activity.

Factor	Effect	Sum of squares	*p*	% contribution
*A*, culture media volume	−0.63	1.17	**0.0264**	5.81
*B*, CuSO_4_	0.80	1.90	**0.0107**	9.39
*D*, glucose	0.17	0.09	0.4304	0.44
*E*, NH_4_SO_4_	0.15	0.07	0.4800	0.35
*F*, peptone	0.62	1.17	**0.0267**	5.76
*G*, yeast extract	−0.73	1.60	**0.0149**	7.92

Significant values are in bold.
